# Modifiable Individual Factors Associated with Breastfeeding: A Cohort Study in China

**DOI:** 10.3390/ijerph16050820

**Published:** 2019-03-06

**Authors:** Xialing Wu, Xiao Gao, Tingting Sha, Guangyu Zeng, Shiping Liu, Ling Li, Cheng Chen, Yan Yan

**Affiliations:** Department of Epidemiology and Health Statistics, Xiangya School of Public Health, Central South University, No. 110 Xiangya Road, Kaifu District, Changsha 410078, China; wuxl9404@163.com (X.W.); 18670321975@163.com (X.G.); tingtingsha@csu.edu.cn (T.S.); 15873124652@139.com (G.Z.); liuship92@163.com (S.L.); 18674824070@163.com (L.L.); chengchen_8493@126.com (C.C.)

**Keywords:** breastfeeding, infant, modifiable individual factors, cohort study

## Abstract

Breast milk is an ideal first food for infants in the initial months of life. This study aimed to identify modifiable individual factors in breastfeeding during the first six months of life in Hunan. This birth cohort study was conducted in three communities in Changsha, Hunan province. Data were collected through maternal health manuals and self-administered questionnaires during the follow-up period. To find risk factors and calculate odds ratios, generalized estimating equation models were applied. The final study included 951 mother-infant pairs. The prevalence of exclusive breastfeeding and any breastfeeding in this study was below the World Health Organization’s breastfeeding goals. Infant age, passive smoking after delivery, minor postnatal depression, and feeding-bottles were shown to act negatively on both exclusive breastfeeding and any breastfeeding. In addition, cesarean delivery and delayed breastfeeding initiation had adverse effects on exclusive breastfeeding. Multiparas were less likely to continue any breastfeeding during the first few months. This study highlights the importance of screening probable maternal depression during primary care service and reducing secondhand smoke and feeding-bottle use. The results also suggest that healthcare professionals should provide more assistance and education to multiparas and mothers with cesarean delivery or delayed breastfeeding initiation.

## 1. Introduction

With all the energy and nutrients necessary for the initial months of life, breast milk is an ideal first food for human infants. Compared with formula-fed infants, breastfed infants have lower mortality and infectious morbidity, lower rates of obesity and diabetes, and better cognitive development later in life [1]. Moreover, both mothers who breastfeed and society benefit from breastfeeding [2].

The World Health Organization recommends that women exclusively breastfeed until their babies reach 6 months of age and continue breastfeeding up to 2 years of age or beyond [3]. Despite the widely recognized benefits of breastmilk, globally, only 38% of infants aged 0–6 months are exclusively breastfed, making the identification of modifiable risk factors a high priority [4]. If breastfeeding rates were brought to a universal level, 823,000 child deaths and 20,000 breast cancer deaths could be prevented every year [5]. That formula feeding is popular during the first few months is also a severe and imminent problem facing Chinese infants. A national representative survey conducted in 30 provinces in China has found that rates of exclusive breastfeeding at 6 months were 20.7% [6]. A birth cohort study of 3196 mothers in Ma’anshan, China has shown that only 11.0% of the women exclusively breastfed their infants at 6 months, and the median duration of any breastfeeding was 7.0 months [7].

Risk factors for breastfeeding practices have been explored in numerous studies. According to a Lancet series in 2016, the determinants of breastfeeding practices are a wide range of historical, socioeconomic, cultural, and individual factors [8]. Some of the reported risk factors in Chinese women include a return to employment within 6 months postpartum, a lack of breastfeeding knowledge, younger maternal age, pre-pregnancy obesity, a higher level of education, and delayed breastfeeding initiation [7,9,10,11,12]. However, findings are inconsistent for the effects of cesarean sections and multiparity on breastfeeding [11,13,14,15]. Although passive smoking, postnatal depression, and feeding-bottles have been documented to be harmful to breastfeeding duration in some Western countries [16,17,18], the evidence is not enough in China. 

The purpose of this cohort study is to determine the prevalence of exclusive breastfeeding (EBF) and any breastfeeding (ABF) at 1, 3, and 6 months postpartum, and to identify modifiable individual factors associated with breastfeeding in Hunan province. Meanwhile, the present study included some variables that have received little attention in China, such as probable postnatal depression, passive smoking, and feeding-bottle use.

## 2. Materials and Methods 

### 2.1. Study Design and Setting

This study was based on an in-progress birth cohort, which is being conducted in three communities at Kaifu District of Changsha in Hunan province from January 2015 to December 2020. The present study included 6-month data because the recommended EBF duration is 6 months. The study was approved by the Independent Ethics Committee Institute of Clinical Pharmacology, Central South University, Changsha, China. (Project number: CTXY-130041-3-2).

### 2.2. Sample

From 1 January 2015 to 31 December 2015, a total of 1286 infants were born in the three communities, and the inclusion criteria were: (1) mothers who delivered live-born babies during this period; (2) mothers who were permanent residents in the Kaifu District; (3) healthcare records of infants were registered in the Community Health Management Information System (CHMIS); (4) mothers who had no history of mental illnesses or brain diseases; (5) mothers who agreed to participate in this survey and provided their written informed consents. Eventually, the birth cohort was composed of 976 eligible mother-infant pairs. We excluded twenty-five multiple births, leaving 951 mothers with singleton births in the final analysis.

### 2.3. Measurement

The mother-related variables included maternal age, educational years (≤9 years, >9 years), pre-pregnancy body mass index (BMI) (underweight, normal, overweight, or obese), per capita household income (<2000 Chinese Yuan (CNY), 2000–4999 CNY, 5000–10000 CNY, >10000 CNY), parity (primiparous or multiparous), mode of delivery (vaginal delivery or cesarean delivery), maternal depression at 1 month after childbirth (no or yes), and passive smoking after delivery (no or yes). The infant-related factors were infant gender (male or female), gestational weeks (<37 weeks or ≥37 weeks), birth weight (<2.5 kg or ≥2.5 kg), initiation of breastfeeding (≤1 h or >1 h), prelacteal supplementation (no or yes), and feeding-bottle use at the age of 1 month (no or yes). BMI was calculated as kg/m^2^ and classified into four categories. Mother’s height and weight were extracted from the maternal health manual at the first prenatal care visit. Minor postnatal depression was assessed via the Chinese version of the Edinburgh Postnatal Depression Scale (EPDS), and the cut-off point was defined as 10 or more [19]. Passive smokers were defined as mothers who were exposed to secondhand smoke over 15 min a day after childbirth. Prelacteal supplementation referred to giving newborn babies prelacteal foods or liquids before breastfeeding is established. 

The dependent variables were accordant with the World Health Organization indicators. EBF was defined as breastfeeding without any other food or water [1]. ABF referred to breastfeeding in combination with infant formula, other milk, and/or solid food.

### 2.4. Data Collection

We recruited and trained six medical students from Central South University to collect relevant information about mothers, infants, and families. Data were collected through a self-administered questionnaire that was pre-tested in a pilot study, as well as maternal health manual and CHMIS. Information concerning infant growth, development and feeding practices at 1, 3, and 6 months postpartum were assessed by maternal self-report through telephone interviews. 

### 2.5. Data Analysis

Frequencies were used to describe the characteristics of the study population and the prevalence of EBF and ABF at 1, 3, 6 months postpartum. Generalized estimating equation (GEE) models were applied to find the correlation between outcomes at different follow-up times from the same child and to estimate the association between individual factors and breastfeeding practices. We selected a robust variance estimator, and specified the link function as “logit”, the correlation as “unstructured” and the family as “binomial”. We carried out univariate analyses on each independent variable with EBF and ABF. Considering the lack of sufficient statistical power, variables that were significant at *p* < 0.10 in the univariate analysis were included in the multivariate analysis. The final model only retained independent variables with *p*-values < 0.05 and included observations with no missing data on all covariates. All statistical analyses were performed using SPSS version 22 (IBM, New York, NY, USA).

## 3. Results

Table 1 summarizes the general characteristics of the subjects. The study included 951 mothers of singleton live births. Most participants in the study were aged 25–29 years (82.4%), with a mean age of 29.9 ± 3.9 years, and a total of 903 mothers (96.5%) had more than 9 years of education. Half of the mothers were primipara and 577 (61.1%) women had a vaginal delivery. Approximately 5.5% of the analytic sample had at least probable minor depression at 1-month infant age and 23 (2.5%) exposed to passive smoking after delivery. The numbers of premature infants and low birth weight infants were 32 (3.4%) and 16 (1.7%), respectively. Seventeen percent of the infants used prelacteal supplementation and 63.5% were breastfed within the first hour after birth. At the age of 1 month, 544 (60.1%) babies were fed with bottles.

Of the 951 eligible women, 948 responded to 1 month postpartum breastfeeding questionnaires (response rate 99.7%), 941 (98.9%) responded to 3 month postpartum breastfeeding questionnaires, with 926 (97.4%) of these women responding to the 6 month postpartum breastfeeding questionnaires. The EBF rate was 74.4% and the ABF rate was 96% in the first month. The EBF rates declined from 64.7% at 3 months to 13.8% at 6 months, and the ABF rates decreased from 89.9% at 3 months to 76.6% at 6 months (see Table 2).

Of 2815 observations, 421 had missing data on one or more variables in the final model (see Table 3). Of 2815 observations, 437 had missing data on one or more variables in the final model (see Table 4). Those with or without missing data did not differ with regard to minor postnatal depression, passive smoking, mode of delivery, parity, breastfeeding initiation and feeding-bottle use at 1 month.

Table 3 presents crude and adjusted associations between individual factors and EBF through the GEE approach. After including all significant variables from univariate analysis (*p* < 0.1), six variables remained statistically significant (*p* < 0.05). Mothers who delivered through cesarean (AOR 0.71; 95% CI 0.53, 0.97) were less likely to practice EBF compared to those who delivered vaginally. Mothers who were considered to have minor depressive symptoms were 0.55 times (95% CI 0.32, 0.97) less likely to exclusively breastfeed their infants than mothers without postnatal depression. Mothers who were exposed to secondhand smoke at 1 month postpartum were 0.37 times (95% CI 0.17, 0.82) less likely to maintain EBF than those without passive smoking. Infants who were breastfed after 1 h following parturition were 0.66 times (95% CI 0.48, 0.93) less likely to be exclusively breastfed than infants who were breastfed within 1 h. Moreover, infants with bottle feeding at 1 month were 0.59 times (95% CI 0.45, 0.77) less likely to be exclusively breastfed than those without bottle feeding in the first 6 months of life. Also, EBF practice decreased with an increase in the babies’ ages, AOR 0.64 (95% CI 0.58, 0.70) for 3 months, and AOR 0.05 (95% CI 0.04, 0.06) for 6 months, compared to infants at 1 month old.

Table 4 provides the results of associations between individual factors and ABF. In the final multivariate model, parity, minor postnatal depression, passive smoking, feeding with bottle and infants’ age were retained as important risk factors for ABF. The multiparas group (AOR 0.63; 95% CI 0.44, 0.89) were less likely to practice ABF than the primiparas group. Mothers who were minor depression patients were 0.40 times (95% CI 0.21, 0.75) less likely to carry out ABF than mothers without postnatal depression. Mothers who were passive smokers were 0.27 times (95% CI 0.10, 0.72) less likely to practice ABF than mothers who didn’t inhale secondhand smoke. In addition, infants who were fed with a bottle at 1 month were 0.57 times (95% CI 0.40, 0.83) less likely to continue ABF than those without bottle use. Moreover, ABF practice decreased as babies grew—AOR 0.27 (95% CI 0.19, 0.38) for 3 months, AOR 0.10 (95% CI 0.07, 0.14) for 6 months, compared to infants at 1 month old.

## 4. Discussion

The prevalence of EBF and ABF in this study was below the World Health Organization’s breastfeeding goals. The ABF rate at 6 months (13.8%) differs from that reported in the first Health Service Household Interview Survey of Hunan Province, China in 2013 (44.9%) [20]. It is higher than the rate observed in Anhui Province (11.0%) [7], but is lower than the rate collected from 55 counties in 30 provinces in China (20.8%) [6]. Compared to other countries, the prevalence of EBF at 6 months postpartum in this study is higher than that in Malaysia and India [21], but lower than that in the United States [22]. The findings of this cohort study suggest infant age, passive smoking after delivery, minor postnatal depression, bottle use at 1-month, cesarean delivery, multiparous, and delayed breastfeeding initiation are significant risk factors for breastfeeding practices in the first six months of age in China. 

EBF and ABF were associated with infant age. The proportion of EBF decreased significantly from 64.7% at 3 months to 13.8% at 6 months, which is consistent with studies conducted in Ethiopia and the United States [22,23]. Infant formula and complementary foods might be introduced to infants with the presupposition that human milk alone will not satisfy the need of infants as they approach 6 months of age, which could contribute to early weaning.

In our study, self-reported exposure to passive smoking after delivery was negatively associated with both EBF and ABF. The prevalence rates of maternal smoking (e.g., 1.0% during pregnancy) were low in this study, which may explain the reduced power to detect any association between maternal smoking and breastfeeding. Previous studies have provided evidence that maternal smoking, paternal smoking, and environmental tobacco smoke, during or after pregnancy, are positively associated with the early cessation of breastfeeding [24,25,26,27]. It has been proposed that nicotine increases adrenaline levels, which may lead to vasoconstriction and impaired breast milk ejection and reduce milk production by suppressing prolactin levels [28]. The message about the hazards of passive smoking should be offered to mothers and the public at large.

No previous studies have evaluated the impact of minor postnatal depression on breastfeeding practices in China. Similar to foreign research [18,29,30,31,32], this study observed reduced odds of EBF and ABF for mothers with minor postnatal depression symptoms. The mechanism of effects of maternal depression on breastfeeding is perceived to be multifactorial, including adverse effects on the mother-infant interaction as well as maternal cognition and self-esteem in breastfeeding [33]. However, the relationship remains controversial, as some researchers have reported no association between breastfeeding practices and maternal depression [34,35]. For one thing, the timing of outcome assessments varies greatly in studies that focus on either prenatal or postnatal. Furthermore, several studies lack a clear definition of breastfeeding [34]. Detected depressive symptoms in this study may represent an underlying maternal pathology that was previously neglected, rather than a mental disease directly concerned with postnatal depression. The decreased prevalence of EBF and ABF in women with higher EPDS scores highlights the early recognition and treatment of early postnatal depression. 

The high rate of feeding-bottle users in the first month could be attributed to the fact that this study was done in communities in the provincial capital of Changsha, as Petal et al. [36] reported that high bottle-feeding is found among urban mothers. This cohort study agrees with the findings of other results that bottle use is a significant risk factor for discontinuing breastfeeding [16,31,37]. A mistaken interpretation of the baby’s crying can lead to the introduction of artificial feeding-bottles and reduced breast-sucking and, consequently, lower production of breast milk [37]. This consequence illustrates the significance of an intense campaign and/or interventions to delay the introduction of feeding-bottles to infants. 

The current study discovered that mothers who gave birth naturally were more likely to practice EBF than those who had a cesarean section; this result accords with previous studies [23,38,39,40]. The possible pathological mechanism is divided into two. The first is that cesarean sections may reduce breast milk production and delay the onset time for lactation by disturbing prolactin production. The other is associated with complications of the surgery, which can lead to a long-separated duration of mothers and newborns, and then inhibit mother-infant interaction and early breastfeeding [40]. Nevertheless, there have been different discoveries. A study conducted in mainland China found that cesarean delivery is a positive factor for the duration of EBF [11]. Because of the longer length of hospitalization, more breastfeeding guidance and support from midwives was given to women who had a cesarean section. A systematic review of world literature has asserted that once mothers had initiated breastfeeding before discharge, a cesarean section no longer had significant effects on the breastfeeding rate at 6 months [41]. With the implementation of the two-child policy, China will have another birth peak. The government should take effective action to reduce deliveries by cesarean routes. Prospective women and family members should be informed of the benefits of breastfeeding and the negative effects of cesarean sections on breastfeeding. Health care professionals should provide additional breastfeeding support for women with cesarean sections immediately after childbirth and early postpartum. 

Multiparity represented a risk factor for ABF in our population. However, the effect of parity on breastfeeding remains conflicting. Some studies observed that multiparous mothers had longer breastfeeding durations [14,42], whereas others found a negative association between multiparity and breastfeeding [15]. Some researchers indicate that it is the prior breastfeeding experience, rather than the birth experience, that influences current breastfeeding practices among multiparous mothers [43,44]. A negative or shorter experience may decrease the subsequent breastfeeding duration [43]. Our finding highlights the necessity of giving additional prenatal and postnatal breastfeeding support to multiparous women with no previous breastfeeding experience or shorter previous breastfeeding periods. 

This result agrees with findings in different cultures: delayed breastfeeding initiation predicted a higher risk of discontinuing EBF [45,46,47]. Early breastfeeding initiation contributes to the likelihood of EBF by reducing the use of prelacteal feeds and boosting a mother’s self-efficacy [45]. Health staff should provide adequate skilled support to establish breastfeeding initiation and skin-to-skin contact as soon as possible after delivery [47]. 

To our knowledge, this is one of the first studies investigating the impact of maternal postnatal depression, passive smoking after delivery, and feeding-bottle use at 1 month on breastfeeding practices in China. The study population was representative for the urban population in Hunan. We randomly selected three Streets of the Kaifu District as our study sites. This community-based cohort study was followed up with a relatively large sample size for up to 6 months postpartum. Additionally, we collected data by self-administered questionnaires and adjusted various potential confounders. However, this study has some limitations. A notable limitation is that the self-administered questionnaire was not assessed for validity and might result in some inaccurate information. Secondly, some variables of interest were unavailable, such as pre-delivery breastfeeding intentions, attitudes of mothers and other family members, and the exact duration of breastfeeding. Another limitation is that the precise time when the mothers returned to work was collected in 6 months and remains incomplete. Recall bias was also not controlled in the study; this important bias may have affected the results. In addition, the population only included healthy mothers who live permanently at urban districts of Changsha city, so the conclusions may not generalizable to all Chinese populations. Furthermore, the low prevalence of minor postnatal depression and passive smoking after delivery may be subject to recall bias as these parameters were self-reported by mothers and other objective measures were not used. Feeding-bottle use, moreover, may not interrupt breastfeeding by itself. 

## 5. Conclusions

Our findings broaden the present knowledge of breastfeeding and contribute to improving breastfeeding practices. This study highlights the need to pay special attention to screening probable maternal depression at a primary care service and reducing secondhand smoke and feeding-bottle use. The results also suggest that healthcare professionals should encourage early initiation of breastfeeding and provide more assistance and education to multiparas and mothers with cesarean delivery. Further research is needed to confirm the relationship between these modifiable risk factors and breastfeeding in other areas of China.

## Figures and Tables

**Table 1:** General characteristics of 951 mother-infant dyads.

Characteristics	Frequency (*n*)	Percentage (%)
Per capita household income (CNY)		
<2000	29	3.1
2000–4999	488	53.0
5000–10,000	366	39.7
>10,000	38	4.1
Maternal age group		
<25 years	46	4.9
25–34 years	780	82.4
>34 years	121	12.8
Mother’s education		
≤9 years	33	3.5
>9 years	903	96.5
Pre-pregnancy body mass index		
Underweight	164	18.8
Normal weight	575	65.8
Overweight	103	11.8
Obese	32	3.7
Parity		
Primiparous	480	50.6
Multiparous	468	49.4
Mode of delivery		
Vaginal delivery	577	61.1
Cesarean delivery	368	38.9
Minor postnatal depression		
No	881	94.5
Yes	51	5.5
Passive smoking after delivery		
No	897	97.5
Yes	23	2.5
Infant gender		
Male	492	52.1
Female	452	47.9
Gestational age		
<37 weeks	32	3.4
≥37 weeks	900	96.6
Birth weight		
<2.5 kg	16	1.7
≥2.5 kg	924	98.3
Breastfeeding initiation		
≤1 h	586	63.5
>1 h	337	36.5
Prelacteal supplementation		
No	743	82.6
Yes	157	17.4
Feeding-bottle use		
No	361	39.9
Yes	544	60.1

Note: Frequencies may not equal 951 due to rounding and missing responses for some questions.

**Table 2:** Breastfeeding rates during follow-up.

Infant Age	Total	Exclusive Breastfeeding n (%)	Any Breastfeeding n (%)	Exclusive Formula Feeding n (%)
1 month	948	705 (74.4)	919 (96.9)	29 (3.1)
3 months	941	609 (64.7)	846 (89.9)	95 (10.1)
6 months	926	128 (13.8)	709 (76.6)	217 (23.4)

**Table 3:** Modifiable individual factors affecting exclusive breastfeeding (EBF).

Variables	*N*	Crude OR (95% CI)	Adjusted OR * (95% CI)	*p*-Value
Maternal age	2804	0.97 (0.95, 0.99)	-	-
Mother’s education				
≤9 years	99	1.00 (reference)	-	-
>9 years	2672	1.56 (0.93, 2.61)	-	-
Parity				
Primiparous	1419	1.00 (reference)	-	-
Multiparous	1390	0.80 (0.68, 0.94)	-	-
Mode of delivery				
Vaginal	1704	1.00 (reference)	1.00 (reference)	-
Cesarean	1094	0.72 (0.61, 0.86)	0.71 (0.53, 0.97)	0.029
Minor postnatal depression				
No	2612	1.00 (reference)	1.00 (reference)	-
Yes	149	0.70 (0.47, 1.05)	0.55 (0.32, 0.97)	0.038
Passive smoking after delivery				
No	2660	1.00 (reference)	1.00 (reference)	-
Yes	67	0.53 (0.33, 0.88)	0.37 (0.17, 0.82)	0.014
Breastfeeding initiation				
≤1 h	1730	1.00 (reference)	1.00 (reference)	-
>1 h	1004	0.70 (0.59, 0.84)	0.66 (0.48, 0.93)	0.016
Prelacteal supplementation				
No	2205	1.00 (reference)	-	-
Yes	462	0.69 (0.54, 0.88)	-	-
Feeding-bottle use				
No	1065	1.00 (reference)	1.00 (reference)	-
Yes	1615	0.67 (0.57, 0.78)	0.59 (0.45, 0.77)	0.000
Month				
1 month	948	1.00 (reference)	1.00 (reference)	-
3 months	941	0.64 (0.58, 0.70)	0.64 (0.58, 0.70)	0.000
6 months	926	0.06 (0.05, 0.07)	0.05 (0.04, 0.06)	0.000

Odds ratios from univariate and multiple Generalized Estimating Equation models; OR: odds ratios; CI: confidence interval; AOR: adjusted odds ratios. * Each factor adjusted for the others. The final model only retained independent variables with *p*-values < 0.05 and included observations with no missing data on all covariates, the number in the model = 2394 including 808 women at 1 month, 800 women at 3 months and 786 women at 6 months postpartum.

**Table 4:** Modifiable individual factors affecting any breastfeeding (ABF).

Variables	*N*	Crude OR (95% CI)	Adjusted OR * (95% CI)	*p*-Value
Parity				
Primiparous	1419	1.00 (reference)	1.00 (reference)	-
Multiparous	1390	0.63 (0.47, 0.84)	0.63 (0.44, 0.89)	0.001
Mode of delivery				
Vaginal	1704	1.00 (reference)	-	-
Cesarean	1094	0.73 (0.55, 0.98)	-	-
Gestational age				
≥37 weeks	2667	1.00 (reference)	-	-
<37 weeks	94	0.53 (0.28, 1.01)	-	-
Minor postnatal depression				
No	2612	1.00 (reference)	1.00 (reference)	-
Yes	149	0.38 (0.22, 0.65)	0.40 (0.21, 0.75)	0.005
Passive smoking after delivery				
No	2660	1.00 (reference)	1.00 (reference)	-
Yes	67	0.45 (0.19, 1.08)	0.27 (0.10, 0.72)	0.008
Breastfeeding initiation				
≤1 h	1730	1.00 (reference)	-	-
>1 h	1004	0.70 (0.52, 0.95)	-	-
Prelacteal supplementation				
No	2205	1.00 (reference)	-	-
Yes	462	0.58 (0.41, 0.83)	-	-
Feeding-bottle use				
No	1065	1.00 (reference)	1.00 (reference)	-
Yes	1615	0.53 (0.39, 0.72)	0.57 (0.40, 0.83)	0.003
Month				
1 month	948	1.00 (reference)	1.00 (reference)	-
3 months	941	0.28 (0.20, 0.39)	0.27 (0.19, 0.38)	0.000
6 months	926	0.10 (0.07, 0.15)	0.10 (0.07, 0.14)	0.000

Odds ratios from univariate and multiple Generalized Estimating Equation models; OR: odds ratios; CI: confidence interval; AOR: adjusted odds ratios. * Each factor adjusted for the others. The final model only retained independent variables with *p*-values < 0.05 and included observations with no missing data on all covariates, the number in the model = 2378 including 802 women at 1 month, 795 women at 3 months, and 781 women at 6 months postpartum.
